# Supplementary material for: NEUROD1 Is Required for the Early α and β Endocrine Differentiation in the Pancreas
Source: Int J Mol Sci. 2021 Jun 23;22(13):6713. doi: 10.3390/ijms22136713 (PMC8268837; doi:10.3390/ijms22136713)
Supplement: Supplementary file 1 [file ijms-22-06713-s001.zip › ijms-1260383-SI.pdf]

Supplementary material

Table S1: Quantification of apoptotic cells in the E17.5 pancreas

**2592_control**			**2595_Neurod1CKO**			
Section#	INS+ cells	other cell types		Section#	INS+ cells	other cell types
1	0	1		1	0	0
2	0	0		2	0	0
3	0	0		3	0	0
4	0	0		4	0	0
5	0	0		5	1	0
6	0	0		6	0	0
**2591_control**			**2594_Neurod1CKO**			
Section#	INS+ cells	other cell types		Section#	INS+ cells	other cell types
1	0	0		1	0	0
2	0	4		2	0	0
3	0	1		3	0	0
4	1	0		4	0	0
5	0	0		5	0	0
6	0	0		6	0	0
**2589_control**			**2590_Neurod1CKO**			
Section#	INS+ cells	other cell types		Section#	INS+ cells	other cell types
1	0	0		1	1	0
2	0	2		2	0	3
3	0	0		3	0	0
4	0	0		4	0	0
5	0	0		5	0	2
6	0	0		6	0	0

* Parafin sections from the E17.5 control and *Neurod1CKO* pancreas immunostained for insulin and apoptotic cells (TUNEL) were evaluated. TUNEL positive cells were manually quantified.

Table S2. Primer sequences for qPCR

Arx_F	TTTTCTAGGAGCAGCGGTGT
Arx_R	GGGCCATAGTGGAAAAGAGC
Pdx1_F	CCCCAGTTTACAAGCTCGCT
Pdx1_R	CTCGGTTCCATTCGGGAAAGG
Pax6_F	GTTGTGTGAGTAAAATTCTGGGC
Pax6_R	GAGTCGCCACTCTTGGCTTA
Neurod1_F	ATGACCAAATCATACAGCGAGAG
Neurod1_R	TCTGCCTCGTGTTCCTCGT
MafA_F	CTCCAGAGCCAGGTGGAG
MafA_R	GTACAGGTCCCGCTCCTTG
MafB_F	GCAGGTATAAACGCGTCCAG
MafB_R	TGAATGAGCTGCGTCTTCTC
Neurog3_F	AGTGCTCAGTTCCAATTCCAC
Neurog3_R	CGGCTTCTTCGCTTTTTGCTG
Pou3f4_F	CTGCCTCGAATCCCTACAGC
Pou3f4_R	CTGCAAGTAGTCACTTTGGAGAA
Ppy_F	CAGGCGACTATGCGACACC
Ppy_R	CAGGGAATCAAGCCAACTGG
Sst_F	ACCGGGAAACAGGAACTGG
Sst_R	TTGCTGGGTTCGAGTTGGC
Gcg_F	CAGAAGAAGTCGCCATTGCC
Gcg_R	AAGTCCCTGGTGGCAAGATT
Ghrl_F	CCAGAGGACAGAGGACAAGC
Ghrl_R	ACATCGAAGGGAGCATTGAA
Ins1_F	GACCAGCTATAATCAGAGACCATC
Ins1_R	GTAGGAAGTGCACCAACAGG
Ins2_F	GGCTTCTTCTACACACCCAT
Ins2_R	CCAAGGTCTGAAGGTCACCT
Nkx2.2_F	AAGCATTTCAAAACCGACGGA
Nkx2.2_R	CCTCAAATCCACAGATGACCAGA
Insm1_F	CTGGCGGCGTATCCGAATC
Insm1_R	CCTGGCGACGGAACTTCTT
Pax4_F	AGGGGGACTCTTTGTGAATGG
Pax4_R	ACCTGTGCGGTAGTAGCGT
Hprt1_F	GTGGCCCTCTGTGTGCTC
Hprt1_R	TCTACAGTCATAGGAATGGATCTATCA
